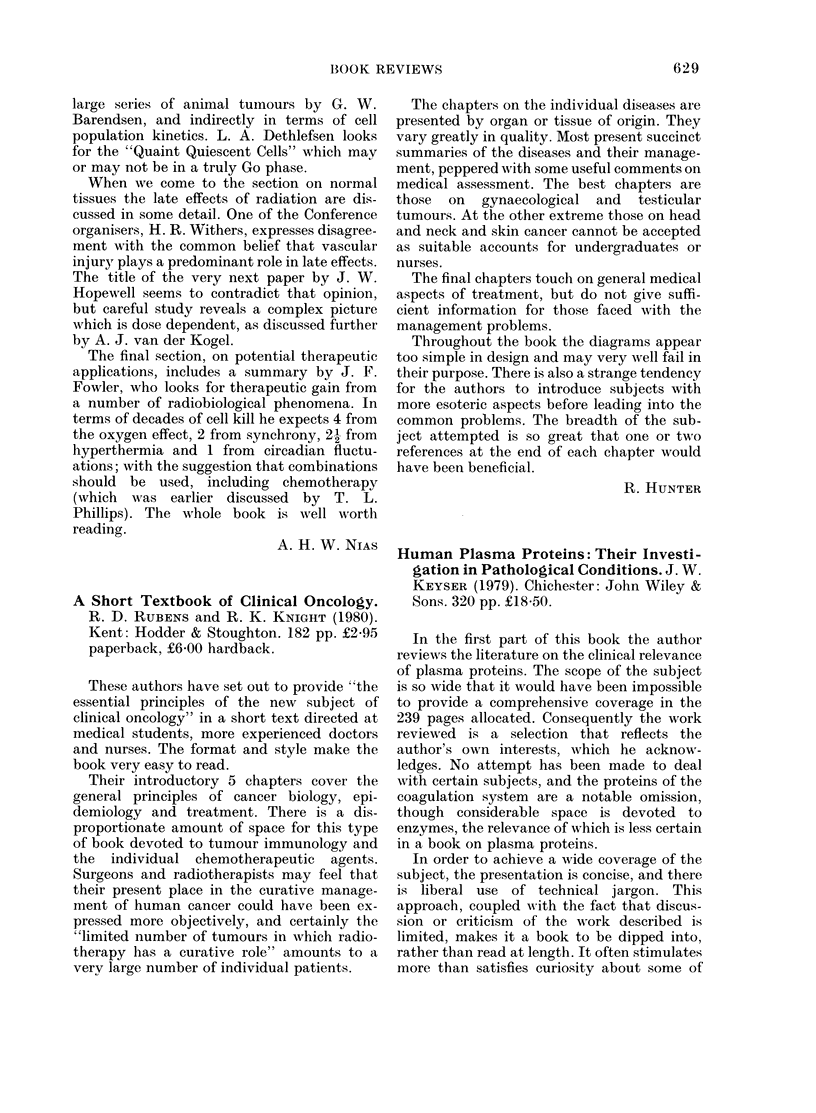# A Short Textbook of Clinical Oncology

**Published:** 1980-10

**Authors:** R. Hunter


**A Short Textbook of Clinical Oncology.** R. D. RUBENS and R. K. KNIGHT (1980). Kent: Hodder & Stoughton. 182 pp. £2·95 paperback, £6·00 hardback.

These authors have set out to provide "the essential principles of the new subject of clinical oncology" in a short text directed at medical students, more experienced doctors and nurses. The format and style make the book very easy to read.

Their introductory 5 chapters cover the general principles of cancer biology, epidemiology and treatment. There is a disproportionate amount of space for this type of book devoted to tumour immunology and the individual chemotherapeutic agents. Surgeons and radiotherapists may feel that their present place in the curative management of human cancer could have been expressed more objectively, and certainly the "limited number of tumours in which radiotherapy has a curative role" amounts to a very large number of individual patients.

The chapters on the individual diseases are presented by organ or tissue of origin. They vary greatly in quality. Most present succinct summaries of the diseases and their management, peppered with some useful comments on medical assessment. The best chapters are those on gynaecological and testicular tumours. At the other extreme those on head and neck and skin cancer cannot be accepted as suitable accounts for undergraduates or nurses.

The final chapters touch on general medical aspects of treatment, but do not give sufficient information for those faced with the management problems.

Throughout the book the diagrams appear too simple in design and may very well fail in their purpose. There is also a strange tendency for the authors to introduce subjects with more esoteric aspects before leading into the common problems. The breadth of the subject attempted is so great that one or two references at the end of each chapter would have been beneficial.

R. HUNTER